# Case 2/2016 - Scimitar Sign with Right Pulmonary Vein Drainage into the Right Atrium

**DOI:** 10.5935/abc.20160023

**Published:** 2016-02

**Authors:** Edmar Atik, Raul Arrieta, Roberto Kalil Filho

**Affiliations:** Hospital Sírio Libanês, São Paulo, SP - Brazil

**Keywords:** Scimitar Syndrome / surgery, Pulmonary Veins / abnormalities, Radiography, Thoracic, Cardiac Catheterization

**Clinical data:** the characteristic signs of the scimitar syndrome with right pulmonary hypoplasia were discovered in an asymptomatic patient through routine chest X-ray, in the presence of dengue.

On physical examination, the patient was in good general health status, eupneic, normal skin color, with normal pulses. His weight was 54 kg, height 155 cm, blood pressure of 100/60 mmHg, heart rate of 88 bpm.

The aorta was not palpable at the sternal notch. In the precordium, there were mild impulses at the left and right sternal borders and the apex beat was not palpable. Heart sounds were normal, with constant splitting of the second heart sound, with discreet and rough ejection systolic murmur in the pulmonary area.

The liver was not palpable and in the lungs, breath sounds were less audible in the right lower pulmonary lobe.

## Complementary tests

**Figure 1:**
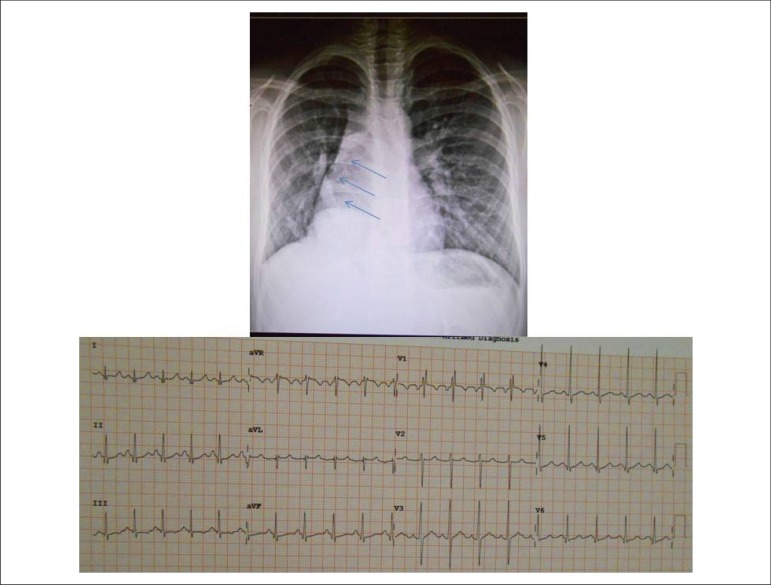
Chest X-ray showed heart dextroposition due to right lung hypoplasia and the scimitar sign (arrows) of the dilated and anomalous right pulmonary vein. Electrocardiogram showed the classic signs of right ventricular volume overload with rSR' complex in V1.

**The Electrocardiogram** showed sinus rhythm and signs of final conduction disturbance in the right branch with rSr' complex in V1. There were no signs of cavity overload. AP: +70, AQRS: + 80º, AT: + 10 (Figure 1).

**The Chest X-ray** disclosed right lung hypoplasia, dextroposition of the heart as a result of it and the classic sign of anomalous pulmonary vein drainage to the right, with scimitar aspect. The pulmonary vasculature to the left was slightly more prominent (Figure 1).

**The Echocardiogram** showed enlargement of the right heart chambers, pulmonary arteries and right pulmonary vein drainage into the right atrium at its lower portion, near the inferior vena cava.

**Figure 2:**
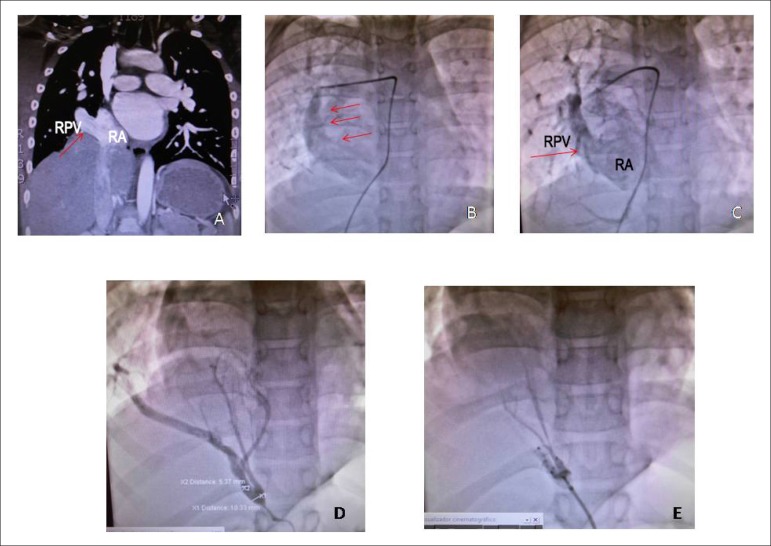
CT angiography in A showing the right pulmonary vein (RPV, arrow) draining into the right atrium (RA); angiography shows the scimitar sign (arrows) of the RPV in B and draining into the RA in C; systemic-pulmonary collateral vessel emerging from the descending aorta into the right lower lobe (pulmonary sequestration) in D and after its embolization in E.

**The CT angiography** disclosed the same aspect, in addition to the obvious systemic-pulmonary collateral circulation from the descending aorta into the right lower pulmonary lobe (Figure 2).

**Diagnosis:** Scimitar syndrome with right pulmonary vein drainage into the right atrium with moderate repercussion and pulmonary sequestration in systemic-pulmonary circulation of the descending aorta into the right lower lobe.

**Clinical reasoning:** the scimitar syndrome, due to the anomalous right pulmonary vein drainage, is clinically manifested as a simple atrial septal defect, with the described classic signs, few symptoms, ejection murmur in the pulmonary area, splitting of the second heart sound and right ventricular diastolic overload on the electrocardiogram. The scimitar sign on the chest X-ray easily characterizes the diagnosis of the syndrome, as this diagnosis had been established in this case. Hence, the importance of this complementary radiographic assessment, simple and definitive, to achieve a diagnostic conclusion of this defect.

**Differential diagnosis:** the scimitar syndrome shows no diagnostic difficulties in comparison to other defects, as it has a characteristic and unique radiographic sign.

**Conduct:** the diagnostic confirmation by cardiac catheterization was scheduled, in addition to the embolization of the systemic-pulmonary vessel, and subsequently, the surgical repair of the anomalous right pulmonary vein drainage was performed. The hemodynamic study showed normal pressures in the heart chambers and arteries (RA = 8, RV = 25/8, PT = 25 / 15-20, Ao = 98 / 58-70 mmHg). Arterial saturation was 100% in the aorta. The angiography showed a large pulmonary venous vessel to the right, quite dilated, which drained into the low right atrium. The injection of contrast into the descending aorta showed an arterial vessel going into the right lower lobe after mild stenosis in its proximal third. It was totally occluded after the placement of three coils (Figure 2).

At the cardiac surgery, after starting the cardiopulmonary bypass, the interatrial septum was partially resected and the right pulmonary vein flow was redirected into the left atrium using a bovine pericardium patch.

The postoperative course was uneventful, with disappearance of the heart murmur.

**Comments:** In classic scimitar syndrome, it is known that drainage of the anomalous pulmonary veins of the right lung in the shape of a scimitar (curved Turkish sword) is directed into the inferior vena cava, with a higher or lower degree of right lung hypoplasia, with or without pulmonary sequestration by the systemic-pulmonary vessel of the aorta to the right lower lobe, as well as dextroposition of the heart. ^1,2^ Most cases are not associated with other defects (75%) and this syndrome shows two different types: the one identified in children (with dynamic repercussions) and the adult type (with less impact of volume overload, related to the degree of right pulmonary hypoplasia). However, the drainage into the right atrium of the right pulmonary veins, while maintaining the scimitar shape is little known. After searching the literature since 1966, we did not find any similar cases to the one described here, with drainage of the anomalous vein directly into the right atrium and the classic scimitar sign. This peculiarity of the scimitar shape persists due to the proximity of the drainage in the lower right atrium, near the inferior vena cava. In this context, there have been reported cases of the scimitar sign, but with normal drainage of the right pulmonary vein into the left atrium itself.^3^ Thus, we currently know the "characteristic scimitar syndrome" and the "scimitar sign" - the latter not associated with the anomalous pulmonary vein drainage or associated with anomalous drainage at another site, such as in the right atrium, for instance.

## References

[1] Vida VL, Padalino MA, Boccuzzo G, Tarja E, Berggren H, Carrel T (2010). Scimitar syndrome: a European Congenital Heart Surgeons
Association (ECHSA) multicentric study. Circulation.

[2] Midyat L, Demir E, Askin M, Gülen F, Ulger Z, Tanaç R (2010). Eponym. Scimitar syndrome. Eur J Pediatr.

[3] Holt PD, Berdon WE, Marans Z, Griffiths S, Hsu D (2004). Scimitar vein draining to the left atrium and a historical review
of the scimitar syndrome. Pediatr Radiol.

